# Supplementary figures and images for: Dissecting the factors shaping fish skin microbiomes in a heterogeneous inland water system
Source: Microbiome. 2020 Jan 31;8:9. doi: 10.1186/s40168-020-0784-5 (PMC6995075; doi:10.1186/s40168-020-0784-5)

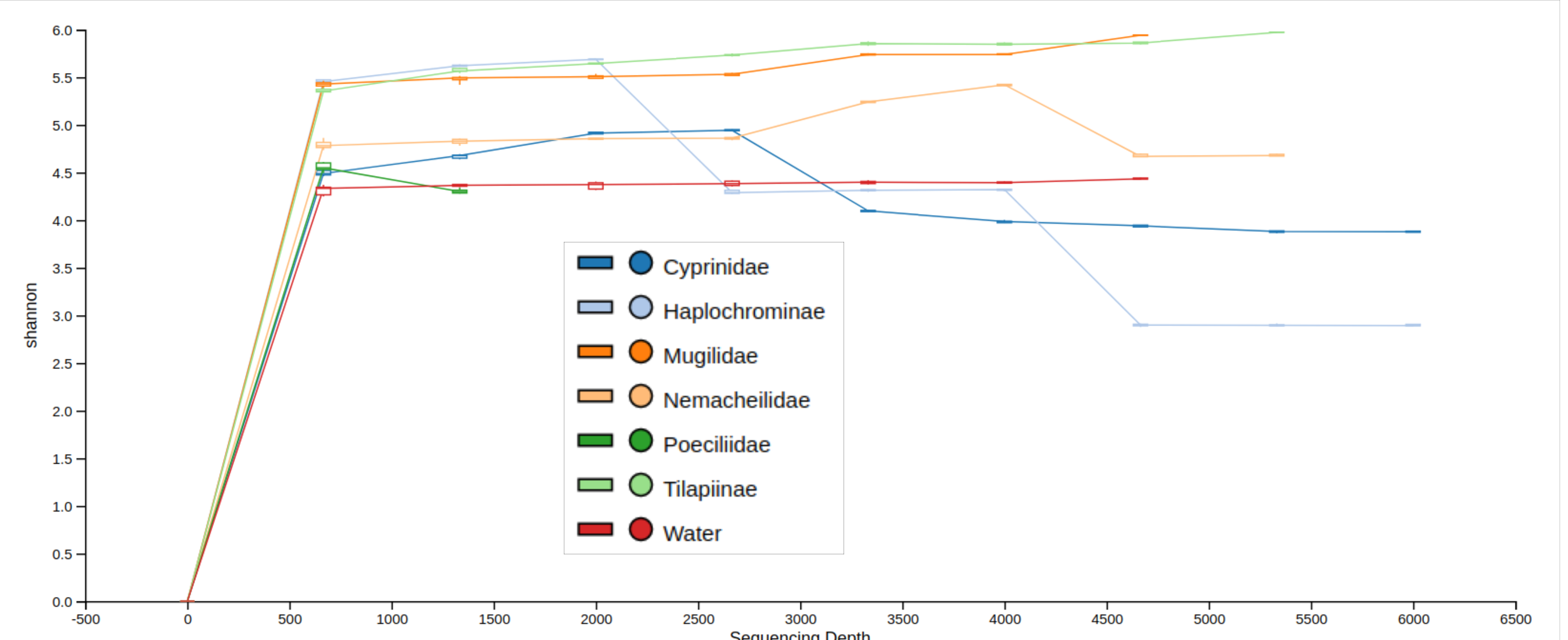

Supplement: Supplementary file 2 — Additional file 1: Figure S1. Alpha rarefaction curves for each fish family or tribe, denoted by the color legend. The x axis is the size of sequence reads subsample and the y axis is the Shannon diversity in the subsample. [file 40168_2020_784_MOESM1_ESM.png]

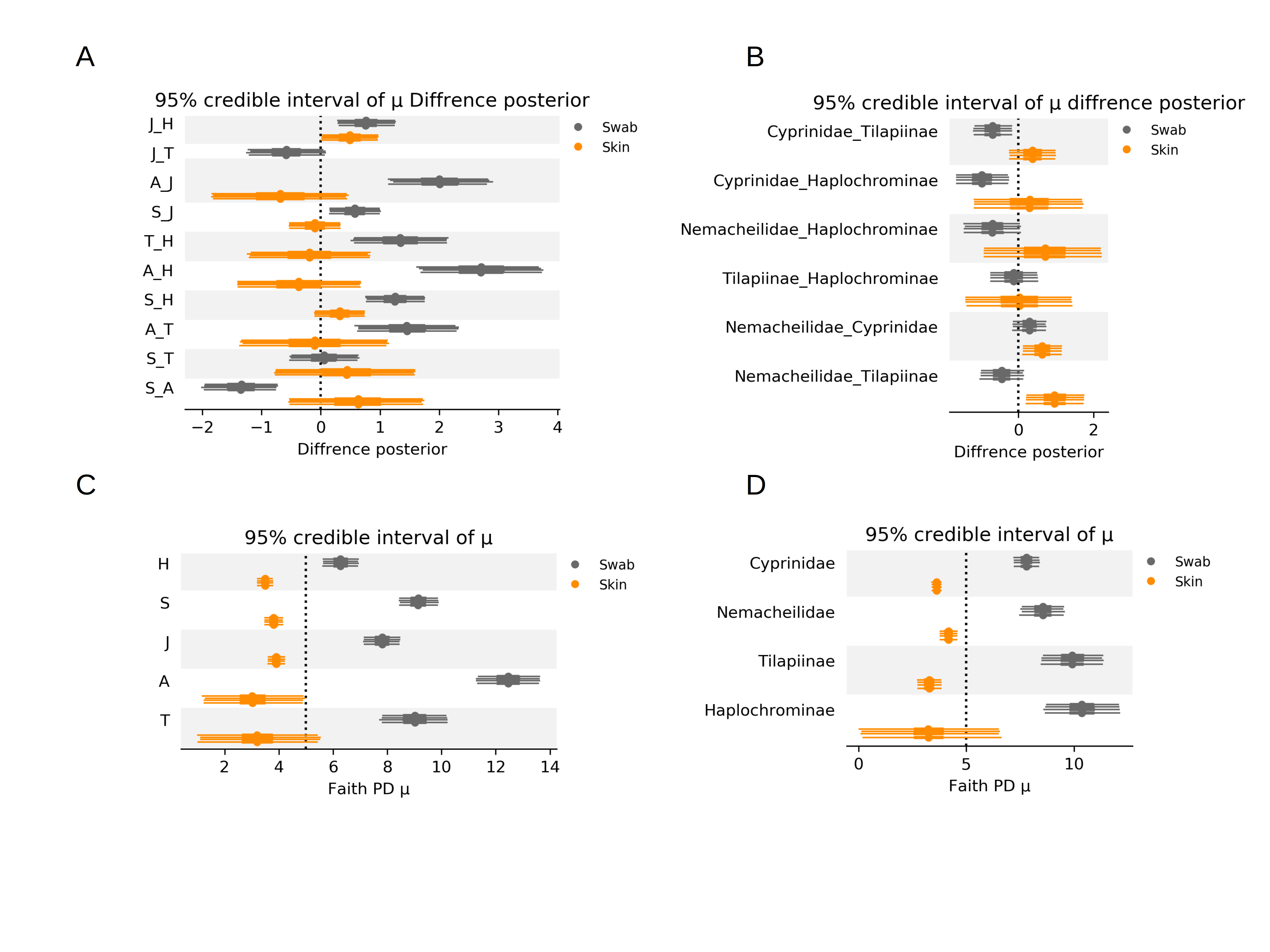

Supplement: Supplementary file 3 — Additional file 2: Figure S2. Posterior distributions of μ pairwise differences (A and B) and of μ (C and D) in streams and fish families. Each confidence interval represent one of four posterior sampling chains that were carried out for each comparison or for each level. Grey intervals represent the raw swab samples and the orange interval represent the skin corrected samples. [file 40168_2020_784_MOESM2_ESM.png]

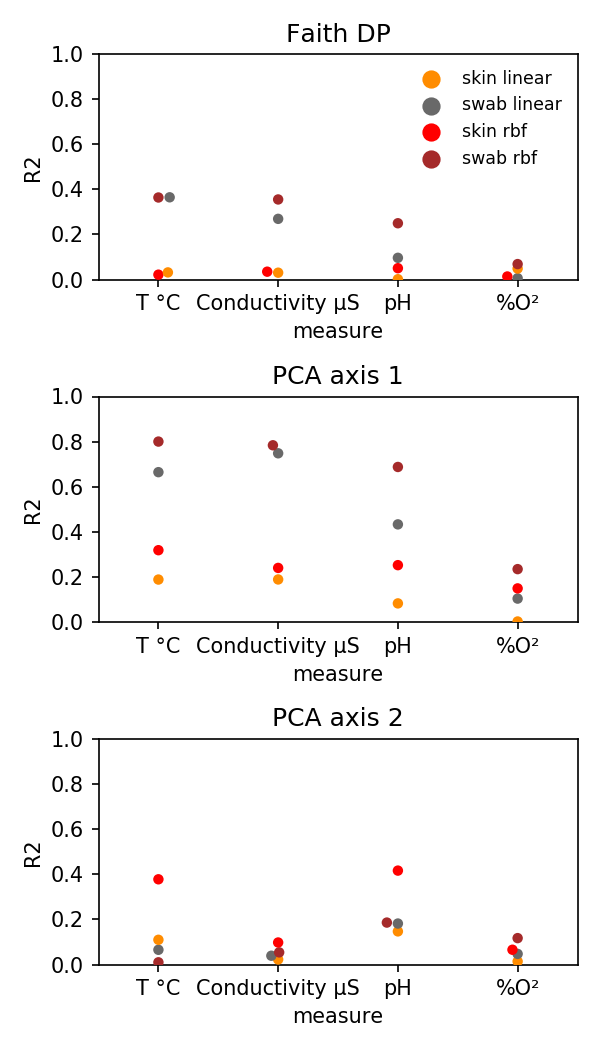

Supplement: Supplementary file 4 — Additional file 3: Figure S3. A comparison of R2 values between approaches with and without assumptions of linear relationships. To evaluate the effect that assumptions of linear relationships have on the proportion of principal component values that is explained by environmental factors, we compared the R2 values that were obtained with a linear kernel with those obtained with an RBF kernel. This was carried out with kernel PCA followed by SVR between PC1 or PC2, and one of the water physicochemical measurements. [file 40168_2020_784_MOESM3_ESM.png]
